# Species-Specific Relationships between Water Transparency and Male Coloration within and between Two Closely Related Lake Victoria Cichlid Species

**DOI:** 10.1155/2012/161306

**Published:** 2012-07-19

**Authors:** Ruth F. Castillo Cajas, Oliver M. Selz, Erwin A. P. Ripmeester, Ole Seehausen, Martine E. Maan

**Affiliations:** ^1^Theoretical Biology Group, Centre for Ecological and Evolutionary Studies (CEES), University of Groningen, P.O. Box 11103, 9700 CC Groningen, The Netherlands; ^2^Department of Fish Ecology and Evolution, Eawag Centre of Ecology, Evolution and Biogeochemistry, Seestraße 79, 6047 Kastanienbaum, Switzerland; ^3^Department of Aquatic Ecology, Institute of Ecology and Evolution, University of Bern, Baltzerstraße 6, CH-3012 Bern, Switzerland; ^4^Sylvius Laboratory, Behavioural Biology, IBL, Leiden University, P.O. Box 9505, 2300 RA Leiden, The Netherlands; ^5^Behavioural Biology Group, Centre for Behaviour and Neurosciences, University of Groningen, P.O. Box 11103, 9700 CC Groningen, The Netherlands

## Abstract

Environmental variation in signalling conditions affects animal communication traits, with possible consequences for sexual selection and reproductive isolation. Using spectrophotometry, we studied how male coloration within and between populations of two closely related Lake Victoria cichlid species (*Pundamilia pundamilia* and *P. nyererei*) covaries with water transparency. Focusing on coloration patches implicated in sexual selection, we predicted that in clear waters, with broad-spectrum light, (1) colours should become more saturated and (2) shift in hue away from the dominant ambient wavelengths, compared to more turbid waters. We found support for these predictions for the red and yellow coloration of *P. nyererei* but not the blue coloration of *P. pundamilia*. This may be explained by the species difference in depth distribution, which generates a steeper gradient in visual conditions for *P. nyererei* compared to *P. pundamilia*. Alternatively, the importance of male coloration in intraspecific sexual selection may differ between the species. We also found that anal fin spots, that is, the orange spots on male haplochromine anal fins that presumably mimic eggs, covaried with water transparency in a similar way for both species. This is in contrast to the other body regions studied and suggests that, while indeed functioning as signals, these spots may not play a role in species differentiation.

## 1. Introduction

Heterogeneous signaling conditions exert divergent selection on animal communication traits, leading to the divergence of sexual signals between environments [1–3]. For example, bird song characteristics may covary with the sound transmission properties of the vegetation (e.g., [4]) and fish coloration may covary with underwater light conditions (e.g., [5]). These adaptations could contribute to reproductive isolation between populations and possibly promote speciation [6–9]. In addition, signalling conditions may influence the opportunity for sexual selection, by compromising signal perception or by increasing the costs of mate searching [10–12].

 The haplochromine cichlids of East Africa constitute a species-rich assemblage with extensive variation in male coloration. Several lines of evidence suggest that variation in underwater light conditions influences the evolution of these colour patterns. In Lake Victoria, for example, male colours tend to become more distinctive in locations with relatively high water transparency [13, 14] and some colour morphs are completely absent in turbid waters [15].

Haplochromine coloration mediates both intraspecific sexual selection [16, 17] and interspecific behavioural isolation [18–20]. Thus, environment-dependent adaptation in male colours may contribute to reproductive isolation. Indeed, there is a relationship between species diversity and colour diversity along water transparency gradients in Lake Victoria, indicating that constraints on visual communication may explain variation in species richness [13].

**Figure 1:**
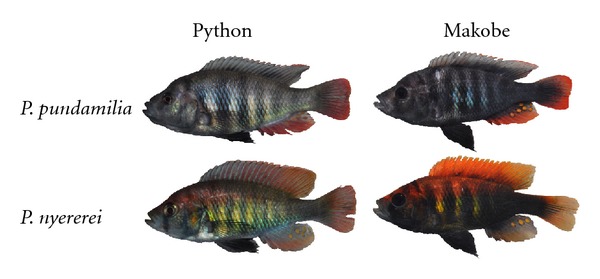
Examples illustrating the colour variation between species and populations.

 Here, we focus on the species pair *P. pundamilia* and *P. nyererei*. These two closely related species are morphologically similar, and the cryptically coloured females of both species are difficult to distinguish. Males however differ markedly in coloration: male *P. pundamilia *are blue-grey while male *P. nyererei* are bright red and yellow (Figure 1). The species co-occur at various locations in Lake Victoria that differ in water transparency.  In the present study, we investigate how this variation in signalling conditions may affect male coloration in both species. Previous work indicated that, within *P. nyererei*, populations inhabiting turbid waters exhibit less red coloration in males [13, 14] and weaker colour preferences in females [14] compared to clear-water populations.

 We address the following predictions. First, we predict that colours are less saturated (i.e., less chromatic) in turbid waters. Since less-saturated colours can reflect a broader range of wavelengths, we expect these to be favoured (i.e., reflect more light and thus be more conspicuous) in turbid water. Second, we assume that colour conspicuousness is constrained by the ambient light intensity at the wavelengths of reflectance. As a result, colours outside the dominant wavelengths of the ambient spectrum will be favoured only in clear waters where their absolute intensities are high enough for receivers to detect. We therefore predict that, in clear water, reflectance should shift towards either shorter (blue) or longer (red) wavelengths, away from the dominant wavelengths (green) in the ambient light.

Finally, as a result of the above changes, we predict that colour differentiation between *P. pundamilia* and *P. nyererei* will be more pronounced in clear waters.

## 2. Methods

### 2.1. Study Species and Sampling Locations

**Figure 2:**
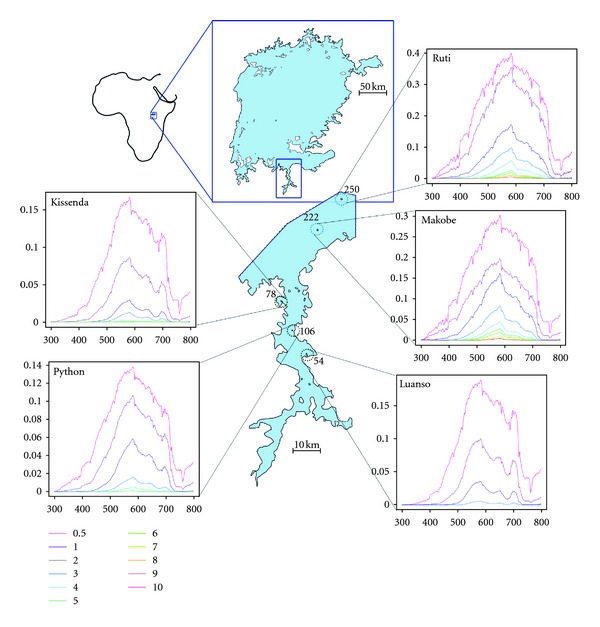
Sampling locations and their underwater light environments. In each panel, curves show underwater ambient light spectra at different depths (m). Numbers shown next to the islands are the mean Secchi disk measurements (cm).

*Pundamilia pundamilia* and *P. nyererei *are two closely related species of haplochromine rock-dwelling cichlids that co-occur throughout a gradient of light environments in Lake Victoria. Both species are morphologically very similar. Females of both species show a yellowish cryptic coloration and are difficult to tell apart. *P. pundamilia* males are blue-grey while males of *P. nyererei* are red dorsally with yellow flanks (Figure 1). Females of both species exert species-assortative colour preferences [18]. In *P. nyererei, *male yellow and red coloration is subject to directional sexual selection as well [14, 17].  Due to its shallow depth, Lake Victoria has relatively turbid waters. Light scattering and absorption are mostly due to nonphytoplankton particles, derived from soil erosion and resuspended sediment [21].  In the present study, we focus on five islands in the south of the lake (Figure 2; Table 1). Two of these islands (Makobe and Ruti) are located offshore and have relatively clear waters. Here, *P. pundamilia* inhabits the top shallower waters while *P. nyererei* dwells in deeper waters. In turbid waters (Kissenda and Python), both species inhabit the same shallow depth layers [22]. At even more turbid locations (i.e., Luanso island), the two species are replaced by a single panmictic population with variable coloration, referred to as *P. *sp*. “Luanso” *[22]. *Pundamilia* sp. breed year-round, with no marked seasonality in breeding activity. All data were collected during May-June 2010.

### 2.2. Underwater Light Environments

At each island, water transparency was measured using a white Secchi disk (Table 1). We measured downwelling irradiance at each island using a BLK-C-100 spectrophotometer and an F-600-UV-VIS-SR optical fiber with CR2 cosine receptor (StellarNet, FL). Measurements were collected in 0.5 m depth increments down to 5 m depth and subsequent 1 m increments down to 12 m depth. At turbid locations, light intensities were too low to obtain reliable measures over this entire depth range (Luanso: measurements down to 4 m; Kissenda and Python: down to 7 m). During each measurement series, we took a minimum of two irradiance spectra at each depth and used the average for further analysis (for repeatability estimates see Supplementary Table S1 in Supplementary Material available online at doi:10.1155/2012/161306). We collected 2 independent measurement series for Luanso island, 3 series each for Kissenda and Ruti islands and 4 series each for Python and Makobe islands (Table 1).

 To characterise variation in light environments between locations and depth ranges we calculated the orange ratio for each spectrum [24, 25]: the light intensity in the 550–700 nm range (yellow, orange, red) divided by the intensity in the 400–550 nm range (blue, green). This ratio reflects the spectral composition of the ambient light and tends to increase with depth and with increasing turbidity, as short wavelengths are selectively scattered and absorbed [22, 26]. We subsequently fitted island-specific exponential curves to obtain estimated orange ratios at each depth. Using the species-specific depth ranges (obtained from [22] and assuming equal distributions at Makobe and Ruti) we subsequently identified the range of orange ratios that each species experiences in its natural habitat.

### 2.3. Reflectance Spectrophotometry

**Figure 3:**
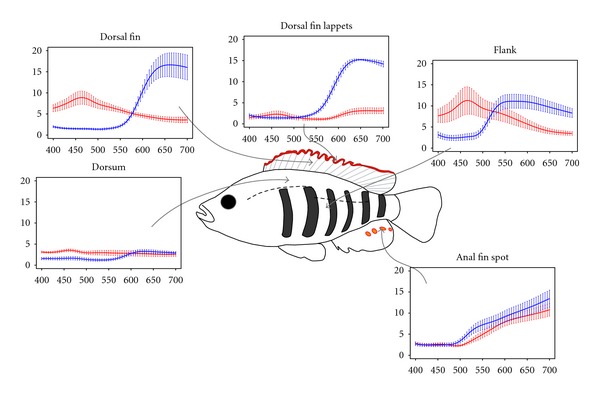
Body areas measured and reflectance spectra for each species (average with standard error; *P. pundamilia* in blue, *P. nyererei* in red; both from Makobe island).

Adult males of the three *Pundamilia* species were collected by gillnetting and angling (sample sizes are given in Table 1). Immediately after collection, reflectance spectra at different areas of the body (Figure 3) were taken using the above-mentioned spectrophotometer, an SL4-DT (Deuterium/Tungsten) light source and an R600-8-UV-VIS reflectance probe (StellarNet, FL). We focused on body parts that are potentially subject to (divergent) sexual selection. In *P. nyererei*, sexually selected coloration (red and yellow; [14, 17]) is mostly present on the flank, dorsum, and dorsal fin. In *P. pundamilia*, intraspecific sexual selection has not been explored and we therefore analysed the same body areas, that are grey-blue in this species. However, red coloration is present also in *P. pundamilia, *namely, on the edges (“lappets”) of the unpaired fins. In order to capture potentially important variation in this trait, we included “dorsal fin lappets” as an additional body area for both species. Finally, for both species we also measured the spectra of the anal fin spots (“egg dummies”) as these brightly coloured spots have been implicated in sexual communication [27–30]. For correlations between body areas, see Supplementary Tables S4 and S5.

About halfway through the field work, the light source stopped working and subsequent measurements had to be taken using the sun as a light source (see below for statistical incorporation of this variation).

### 2.4. Calculation of Colour Metrics

A minimum of two reflectance spectra were measured for each body region for each fish, and the mean of these was used for calculations (unless after visual inspection, one of the spectra was outside expected limits and was discarded, less than 10% of all spectra; repeatability estimates for included spectra are given in Supplementary Tables S2 and S3). We then extracted two colour metrics (see Table 2), excluding the UV part of the spectrum (300–400 nm) because UV-sensitive pigments have not been detected in Lake Victoria cichlids including *Pundamilia* species [31, 32]. (1) Chroma (or saturation): a measure of the purity of a colour, indicating how much of the reflectance is concentrated in a particular segment of the spectrum. It ranges from 0 (e.g., grey or white) to 1 (a pure colour). (2) Hue: related to the wavelength at the maximum absolute slope in the reflectance spectrum, and the property that in common language we understand as colour (e.g., red, blue, green, etc.). As a measure of hue, we calculated *λP*_50_, the wavelength at which 50% of the total reflectance between 400–700 occurs [33, 34].

Brightness, that is, the total intensity of light reflected, is another potentially important component of coloration. However, due to the failure of the light source we did not obtain reliable brightness estimates (see below) and therefore excluded this property from the analyses.

### 2.5. Data Analysis

**Figure 4:**
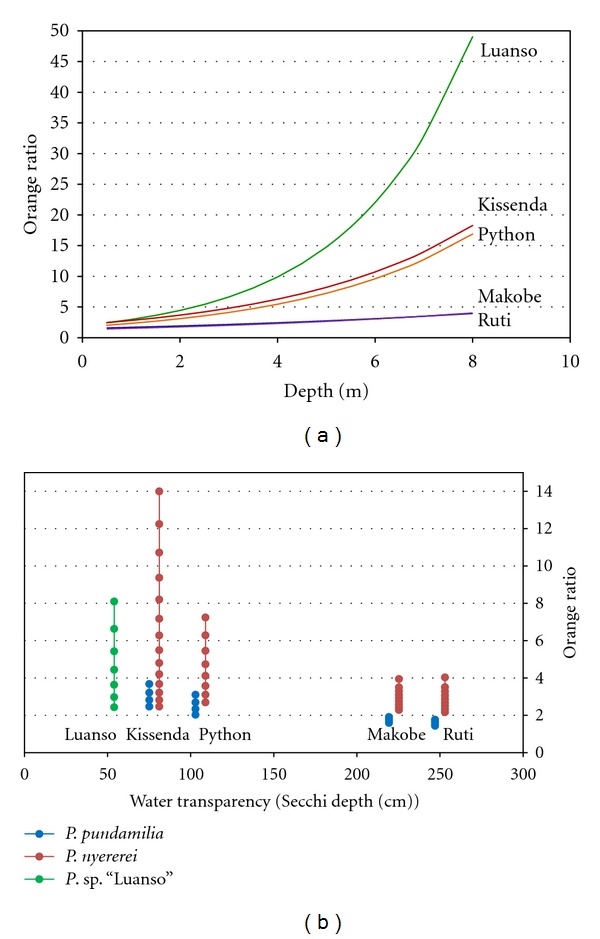
Variation in underwater light environments between sampling locations and species-specific depth ranges. For each location, plotted values derive from fitting an exponential function to all measured orange ratios at that location. (a) The increase in orange ratio with depth for the five sampling locations. Ruti and Makobe show virtually identical curves. (b) The orange ratios at the species- and island-specific depth ranges. Each symbol represents the orange ratio at a specific water depth (in 0.5 m increments) where the species occur.

We built linear models allowing for random effects as well as differences in variances among the explanatory variables, using Linear Mixed Effect models (LME) [36]. We fitted models for each coloration property, each body area, and each species separately. We chose this approach (as opposed to collapsing metrics and body areas into, for example, Principal Components) because it allows evaluation of specific predictions and exposes potential differences between body areas. All analyses incorporated four populations of each species (Luanso was excluded from the analyses but included in the figures as a reference). Because water transparency was bimodal rather than continuous (i.e., the waters at Kissenda and Python islands were similarly turbid, and Makobe and Ruti similarly clear, Table 1; Figure 4), water clarity was modelled as a categorical variable (i.e., turbid versus clear). A factor for population was included as a random effect in all models. In addition to water clarity, the effect of using either the lamp or the sun as a light source was included as explanatory variable. To address colour differentiation between species, species identity was added as a third explanatory variable and the interaction with water clarity evaluated.

For model selection, we explored all possible variance structures (variance components were functions that included the actual Secchi depths (Table 1) and a factor for light source) and selected the most parsimonious model using restricted maximum likelihood ratio and Akaike's information criterion, corrected for small sample size (AICc) [37]. After remaining with the best variance structure, we used maximum likelihood to reduce the complexity of the models and AICc to select the covariates that remain in the model. We then used ANOVA to test whether a model including the clarity covariate (or the interaction between species: clarity, when applicable) was significantly better than one that did not, and we report likelihood ratio and *P* values for this comparison.

All statistical analyses were conducted in R 2.12 [38], applying packages *nlme *and *MuMIn*. To adjust for multiple testing of the same prediction in multiple body areas, we used corrected *P* values (i.e., we multiplied the actual *P* values with the number of body areas, 5).

Our estimates of chroma and hue were not strongly influenced by the light source used (lamp or sun, see Supplementary Table S6) but there were major effects on brightness, showing significant interactions between water clarity and light source for all models. Therefore, we had to discard this metric.

## 3. Results

### 3.1. Light Environments

At all study sites, the proportion of longer wavelengths in the light spectrum (i.e., wavelengths >550 nm) increased towards deeper waters (Figure 4(a)). The increase was steepest at Luanso, intermediate at Kissenda and Python islands, and very gentle at Makobe and Ruti islands. Incorporating species-specific depth ranges at each location, we estimated the range of orange ratios that the two species experience in their natural habitats. Both species are exposed to higher orange ratios in the turbid waters of Kissenda and Python, compared to Makobe and Ruti (Figure 4(b)). *P. nyererei* in particular experiences a large difference in light environment between turbid and clear locations, although the decrease in orange ratio was not significantly different between the species (ANOVA, interaction effect between Secchi reading and species on orange ratio: **F*_2,4_ = 4.49*, *P* = 0.10).

### 3.2. Chroma

**Figure 5:**
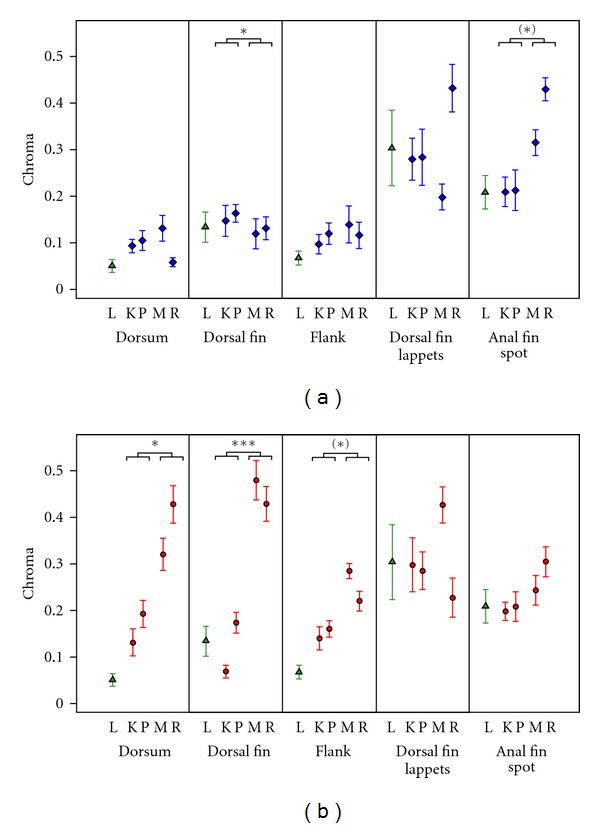
Chroma of different body parts at four sampling locations for (a) *P. pundamilia* (blue diamonds) and (b) *P. nyererei *(red circles). In both panels, *P. *sp*. “Luanso”* (green triangles) is included as a reference. Symbols indicate means with standard errors. Statistically significant differences between clear and turbid locations are indicated with asterisks (after correction for multiple testing; (*) *P* < 0.10; *P < 0.05; ***P < 0.001). L: Luanso, K: Kissenda, P: Python, M: Makobe, R: Ruti.

In *P. pundamilia *(Figure 5(a)), we did not observe a significant increase in chroma in any of the measured body areas. There was a trend for anal fin spots (**L* = 5.66*, *P* = 0.087), but a significant decrease in chroma for dorsal fin (**L* = 6.81*, *P* = 0.045). There were no changes in the chroma of the dorsum, flank, or dorsal fin lappets. The changes in *P. nyererei* were more consistent (Figure 5(b)), with significantly increased chroma in clearwater populations for dorsum (**L* = 9.16*, *P* = 0.013) and dorsal fin (**L* = 12.53*, *P* < 0.001) and a trend in the same direction for flank (**L* = 5.99*, *P* = 0.072). No significant changes were observed in anal fin spots and dorsal fin lappets.

### 3.3. Changes in Hue

**Figure 6:**
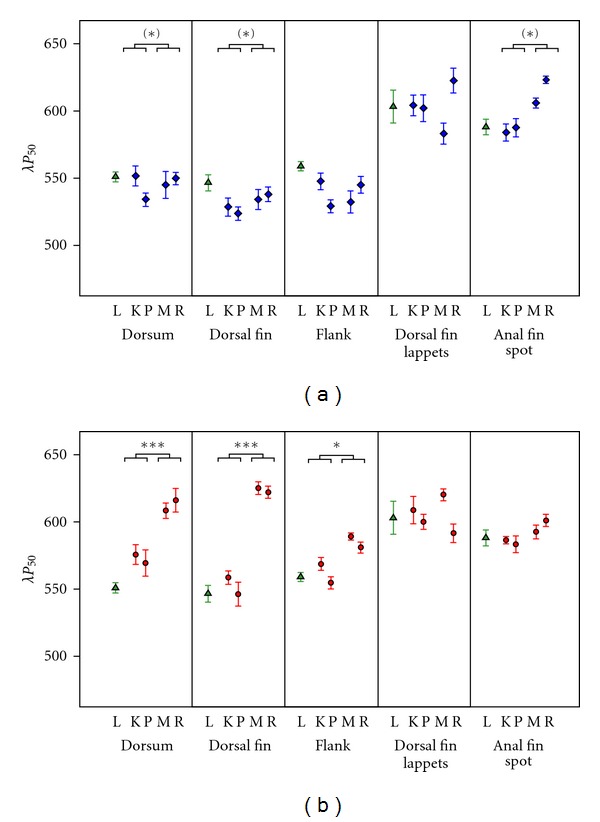
Hue (*λP*_50_) of different body parts at four sampling locations for (a) *P. pundamilia* and (b) *P. nyererei*. Symbols and labels as in Figure 5.

*λP*_50_ (the wavelength that halves the total reflectance) was expected to shift towards more extreme wavelengths in clear waters. For the blue coloration elements in *P. pundamilia*, results were inconsistent with this prediction (Figure 6(a)). We found small and nonsignificant changes towards longer rather than shorter wavelengths for dorsum (**L* = 6.36*, *P* = 0.059) and dorsal fin (**L* = 5.76*, *P* = 0.082). There was no significant change in the hue of flank coloration. The red dorsal fin lappets also did not increase in *λP*_50_. Only the yellow anal fin spots tended to follow the prediction, but the increase towards longer wavelengths in clear water was not statistically significant (**L* = 6.17*, *P* = 0.065).

In *P. nyererei* (Figure 6(b)), we observed a highly significant shift towards longer wavelength reflectance for the dorsum (**L* = 11.51*, *P* < 0.001), dorsal fin (**L* = 15.69*, *P* < 0.001) and flank (**L* = 9.28*, *P* = 0.012). Anal fin spots and dorsal fin lappets did not show significant changes.

### 3.4. Colour Differentiation between Species

**Figure 7:**
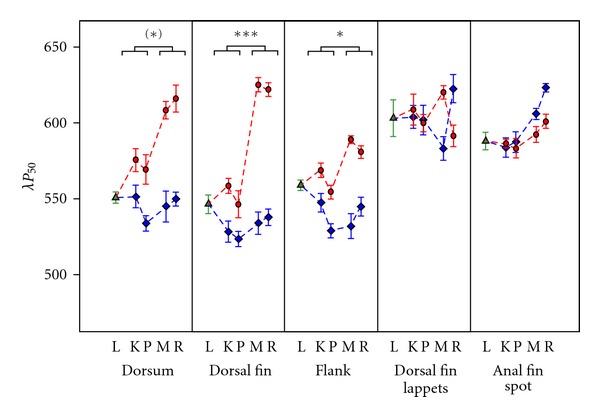
Differentiation in coloration hue (*λP*_50_) of different body regions between *P. pundamilia *and *P. nyererei. *Symbols and labels as in Figure 5.

*λP*_50_ was also used to test for the extent of differentiation between the two species' coloration (Figure 7). We found increased differentiation in clear waters for dorsal fin (**L* = 27.29*, *P* < 0.001), and flank (**L* = 8.77*, *P* = 0.016) and a trend in the same direction for dorsum (**L* = 5.52*, *P* = 0.094). In contrast, coloration of anal fin spots and dorsal fin lappets did not show increased differentiation with water clarity.

## 4. Discussion

We examined patterns of colour variation within and between two cichlid species that inhabit different signalling environments. We specifically tested whether fish coloration becomes more saturated and increasingly exploits wavelength ranges outside the dominant ambient light spectrum, in populations inhabiting clearer waters. We found support for these predictions in *P. nyererei*, but inconsistent results for *P. pundamilia. *For those body areas that are differently coloured between the species, we observed increasing species differentiation in coloration towards clear waters.

 For the red and yellow coloration elements in *P. nyererei*, we found that colours are more saturated and shifted towards longer wavelengths (i.e., redder) in clearer waters. For the blue coloration of *P. pundamilia* however, we did not observe any statistically significant shift towards greater chroma or shorter wavelengths (i.e., more blue). One reason for this incongruence may lie in the different depth distributions of the two species. The change in the environmental light spectrum from turbid to clear waters is more pronounced in the deeper waters where *P. nyererei* is most abundant (Figure 4), possibly generating stronger divergent selection between allopatric populations for this species. It is also possible that the importance of male coloration for intraspecific female choice differs between the species. Sexual selection on red and yellow colour elements is well established in *P. nyererei* [14, 17], but intraspecific sexual selection remains to be studied in *P. pundamilia. *Just like *P. nyererei*,* P. pundamilia* females use colour cues during interspecific mate choice [18]. However, they might use other characteristics, such as body size, behaviour or chemical cues in their choice among conspecific males. Recent work in these and other haplochromines indicates that chemical cues could play a role in mate choice in some species [39–41]. Methodological constraints may also contribute to the difference between species, as the blue-grey coloration of *P. pundamilia* may be more difficult to capture with spectrophotometry [42]. This is consistent with the observation that the yellow anal fin spots did tend to change in the predicted direction for both hue and chroma.

 Although not statistically significant, we observed similar variation in anal fin spot coloration in both species. This is consistent with earlier suggestions of adaptation of these spots to environmental light: Goldschmidt [29] found that species inhabiting darker habitats had relatively large anal fin spots. Anal fin spots have been suggested to mimic eggs and contribute to fertilisation success (e.g., [43] but see [44]). This functional context raises the question whether the observed variation in spot coloration influences the resemblance to eggs. *Pundamilia *sp. eggs are orange, but no data exist regarding egg colour variation between species or populations. Anal fin spots have also been suggested to play a role in speciation (e.g., [30, 45–48]. Here, we do not find evidence for species-specific effects in spot coloration and a role in species recognition is thus unlikely.

 We found no consistent changes in the coloration of the red dorsal fin lappets in either species. Interestingly, this trait is shared not only between our study species, that are very closely related, but also occurs in many other haplochromines [49]. This may indicate that there is little genetic variation in this trait, preventing adaptive divergence between populations and species.

We propose that the differences in coloration that we observed across the four studied populations are adaptations to different underwater light environments. Fish coloration can be phenotypically plastic [50, 51] and in haplochromines, colour expression varies with diet, territorial status [52, 53], and stress ([54]; pers. obs.). However, given the maintenance of colour differences in the laboratory, and significant genetic differentiation between populations [22], evolutionary adaptation is both feasible and likely. We hypothesise that the observed patterns are driven by selection for signal conspicuousness, which requires that signals have sufficient intensity as well as provide contrast against the sensory background [2].

Colour signals that rely on reflection of incident light (as opposed to luminescence or iridescence) will maximise signal intensity by reflecting most strongly in the wavelength range of the incident light (e.g., [55, 56]). However, maximising colour contrast requires reflectance of wavelengths that are underrepresented in the background (e.g., [57]). When the illuminating and background spectra are similar, signal evolution will likely reflect a tradeoff between signal intensity and contrast. This situation occurs in many aquatic systems, where signals are viewed against the water column [58]. In some fish species, conspicuousness is achieved by reflectance of colours that contrast against the prevalent ambient light (e.g., [59, 60]). In other species, colour variation is positively correlated with the prevalence of the reflected wavelengths in the environmental light spectrum [5, 50, 61]. The patterns we observe in *Pundamilia* may reflect a compromise between these two strategies. The blue *P. pundamilia *are restricted to shallow waters where short wavelengths are still present, whereas the red and yellow *P. nyererei *inhabit deeper waters with red-shifted ambient light. At the same time, colour contrast against the background can be maintained by exploiting the shoulders rather than the peak of the ambient spectrum and by reflecting in a relatively narrow wavelength range. We hypothesise that this explains the shift in hue and chroma in the clearwater populations of *P. nyererei*, that experience a broader and more intense illumination spectrum than their counterparts in turbid waters. The failure of our light source precluded analysis of brightness variation in the present dataset. As a consequence, we are unable to test whether the conspicuousness of male coloration is optimised for local viewing conditions. Moreover, recent studies suggest that there is variation in visual systems between sympatric species and allopatric populations of *Pundamilia *[22, 31], and ongoing work is aimed at identifying the visual pigments and expression levels in the populations studied here. This information will subsequently be incorporated into quantitative visual models.

Different patterns of variation may also result from other factors than intraspecific perceptual processes. For example, colour production may be subject to physiological constraints [62, 63]. The red and yellow coloration in *Pundamilia *is carotenoid based [14] and the availability of dietary carotenoids may covary with underwater light intensity [64, 65]. Thus, redder coloration in clearer waters could be due to greater availability of carotenoids. Observations that colour variation between populations is maintained in the laboratory indicate a heritable component, but this does not rule out that carotenoid limitation selectively favours different levels of colour expression [66–68]. Testing this hypothesis requires evaluating whether haplochromines are carotenoid limited in their natural habitat. Second, sexually selected traits are often subject to increased predation (e.g., in fish: [69–72]). In Lake Victoria, however, piscivorous birds and fish tend to be more numerous in clearwater locations [13]; pers. obs), possibly because turbidity hampers visual predation [12, 73]. This would favour less chromatic and less contrasting colours in clearwater, which is not what we observe in *Pundamilia*. Finally, male colour evolution will likely reflect variation in female preferences among populations. Relaxed sexual selection on visual signals in turbid water has been documented in several fish species [74–76]. In addition to immediate effects of reduced signal perception, variation in water turbidity may lead to heritable changes in female preference behaviour. This seems to be the case in *P. nyererei.* Females from turbid waters are less selective with respect to male coloration, even when tested under broad-spectrum illumination in the laboratory [14]. The observed colour variation across populations might therefore be driven by heterogeneous sexual selection regimes, rather than selection for optimal local conspicuousness. To resolve this question, we need more detailed analyses of variation in female preference and choosiness to establish sexual selection strength for the different aspects of male coloration (hue, chroma), as well as quantitative estimates of visual conspicuousness in relation to these aspects. Such studies should also help to identify the mechanisms underlying preference variation. Beside sensory biases for conspicuous signals, haplochromine female preferences are likely influenced by selection for heritable benefits (e.g., parasite resistance [52]). Thus, if signal conspicuousness in turbid waters is maximised by lower carotenoid deposition, for example, carotenoid-dependent aspects of male coloration may become less informative and therefore less important in mate selection (e.g., [77–79]). We suggest that the interactions between sensory processes and signal content in shaping haplochromine colours constitute an important and rewarding avenue for further study.

Taken together, we found that different body regions and different species show different responses to environmental heterogeneity in visual conditions: divergence at the level of allopatric populations as well as sympatric species (flank, dorsum, dorsal fin), divergence between populations but not species (anal fin spots), or no consistent pattern of change (dorsal fin lappets). Importantly, our findings confirm earlier suggestions that divergent sexual selection is involved in haplochromine species divergence [13, 80], as we found significantly stronger species differentiation towards clear waters for the same body areas that were previously shown to be subject to intraspecific sexual selection in *P. nyererei* [17]. As such, our study implicates species- and habitat-specific selective pressures as well as potential genetic or functional constraints to adaptive divergence and thereby contributes to identifying the traits involved in the buildup of reproductive isolation.

## Supplementary Material

Table S1: Repeatability for irradiance data: orange ratio.
Table S2: Repeatability for reflectance data: chroma.
Table S3: Repeatability for reflectance data: *λ*P50.
Table S4: Spearman correlations between body areas for chroma.
Table S5: Spearman correlations between body areas for *λ*P50.
Table S6: Descriptive statistics for the effect of using different light sources.

## Figures and Tables

**Table 1:** Study site characteristics and numbers of individuals collected.

	Luanso	Kissenda	Python	Makobe	Ruti
Maximum depth of the rock-sand interface (m)^1^	5-6	7-8	7-8	8–12	>13
Mean Secchi transparency (cm, mean ± se)^2^	54 ± 4 (*n* = 9)	78 ± 8 (*n* = 8)	106 ± 7 (*n* = 11)	222 ± 7 (*n* = 88)	250 ± 23 (*n* = 7)
Spectral width (and range, nm) of the light spectrum at 2 m depth and 0.002 W/m^2^ light intensity	195 (497–692)	247 (477–724)	264 (455–719)	366 (362–728)	390 (343–733)
Sampling dates for irradiance spectrophotometry (2010)	29/5, 7/6	17/5, 1/6, 9/6	20/5, 26/5, 4/6, 5/6	22/5, 27/5, 3/6, 10/6	24/5, 31/5, 12/6
Sample size *P. pundamilia *	10^3^	8	10	11	9
Sample size *P. nyererei *		6	16	19	17

^1^Data from [23] and *pers. obs*.

^2^Data collected between 2000 and 2010. Water transparency varies seasonally, but differences between sampling locations are highly consistent (for Secchi readings collected during 2000–2010 at our four sampling sites: anova controlling for sampling date: *F*_3,107_ = 25.41, *P* ≪ 0.0001).

^3^ *P. sp. “Luanso”* replaces both species at this locality.

**Table 2:** Coloration metrics.

Name/description	Formula	Reference
	$C=\sqrt{LM^{2}+MS^{2}}$	
	*LM* = *B*_*R*_ − *B*_*G*_	
	*MS* = *B*_*Y*_ − *B*_*B*_	
	where:	
	$B_{B}=\frac{\sum_{400}^{474}Q(\lambda ,x)}{B}$	
Chroma	$B_{G}=\frac{\sum_{475}^{549}Q(\lambda ,x)}{B}$	[35]
A measure of the “purity” or saturation of a colour; a function of how rapidly intensity changes with wavelength	$B_{Y}=\frac{\sum_{550}^{624}Q(\lambda ,x)}{B}$	
	$B_{R}=\frac{\sum_{625}^{700}Q\left(\lambda ,x\right)}{B}$,	
	$B=\sum_{400}^{700}Q\left(\lambda ,x\right)$	

*λP*_50_		
Wavelength that divides the spectrum in two parts with equal spectral energy (i.e., the median of the cumulative distribution between 400–700 nm)	$\sum_{400}^{\lambda P_{50}}\;=\sum_{\lambda P_{50}}^{700}\;$	[33, 34]
