# Supplementary material for: Species-Specific Relationships between Water Transparency and Male Coloration within and between Two Closely Related Lake Victoria Cichlid Species
Source: Int J Evol Biol. 2012 Jul 19;2012:161306. doi: 10.1155/2012/161306 (PMC3408676; doi:10.1155/2012/161306)
Supplement: Supplementary file 1 — Table S1: Repeatability for irradiance data: orange ratio. Table S2: Repeatability for reflectance data: chroma. Table S3: Repeatability for reflectance data: λP50. Table S4: Spearman correlations between body areas for chroma. Table S5: Spearman correlations between body areas for λP50. Table S6: Descriptive statistics for the effect of using different light sources. [file 161306.f1.pdf]

Supplementary Information

Species-specific relationships between water transparency and male coloration within and between two closely related Lake Victoria cichlid species

Ruth F. Castillo Cajas, Oliver M. Selz, Erwin A. P. Ripmeester, Ole Seehausen and Martine E. Maan

Supplementary Table S1: Repeatability for irradiance data: orange ratio

	Luanso			Kissenda			Python			Makobe			Ruti		
depth	N dates	N spectra	r	N dates	N spectra	r	N dates	N spectra	r	N dates	N spectra	r	N dates	N spectra	r
0.5	2	4	0.96	3	6	0.88	4	9	0.99	3	5	0.70	3	5	0.96
1	2	4	0.71	3	6	0.59	4	8	1.00	4	9	0.82	3	5	0.87
1.5	2	4	0.98	3	6	0.67	4	10	1.00	3	6	0.31	3	8	-0.67
2	2	6	0.71	3	8	0.67	4	10	1.00	3	7	-0.16	3	6	0.77
2.5	2	4	0.99	3	6	0.94	4	10	1.00	3	10	0.59	3	6	0.95
3	2	8	0.93	3	8	0.93	4	10	1.00	4	11	0.92	3	6	0.99
3.5	2	7	0.65	3	10	0.91	4	15	1.00	2	5	0.96	3	5	0.82
4				3	6	0.96	4	8	1.00	4	13	0.81	3	8	0.99
4.5				3	8	0.97	4	8	1.00	2	5	0.98	3	6	1.00
5				3	6	1.00	4	8	1.00	4	9	0.98	3	6	1.00
6				2	6	-0.28	3	6	1.00	4	12	0.86	3	8	1.00
7										4	11	0.96	3	6	1.00
8										4	11	0.37	3	8	0.68
9										4	12	0.99	3	6	1.00
10										4	16	-0.29	2	8	0.97
11										4	9	1.00	2	5	1.00
12										3	6	1.00	2	4	1.00

Supplementary Table S2: Repeatability for reflectance data: chroma														
		light source: lamp						light source: sun						
		P. nyererei			*P. pundamilia*			*P. nyererei*			*P. pundamilia*			
		r	N fish	N spectra	r	N fish	N spectra	r	N fish	N spectra	r	N fish	N spectra	
dorsum	Kissenda	NA	1	2	NA	1	2	0.99	5	10	0.63	7	15	
	Python	0.91	9	21	0.63	5	13	0.94	7	13	0.71	5	10	
	Makobe	0.33	9	22	0.61	5	10	0.82	9	20	0.85	4	8	
	Ruti	0.90	11	24	0.57	8	16	0.65	6	15	NA	1	2	
	All islands	0.86	30	69	0.64	19	41	0.90	27	58	0.66	17	35	
dorsal fin	Kissenda	NA	1	2	NA	1	2	0.97	5	9	0.32	7	14	
	Python	0.54	9	20	0.76	4	12	0.74	7	13	0.68	5	10	
	Makobe	0.96	9	18	0.87	5	12	0.85	7	15	0.64	5	9	
	Ruti	0.95	11	22	0.98	8	16	0.93	6	14	NA	1	2	
	All islands	0.94	30	62	0.92	18	42	0.95	25	51	0.58	18	35	
dorsal fin lappets	Kissenda	NA	1	2	NA	1	2	0.83	5	9	0.60	7	13	
	Python	0.91	8	16	0.90	4	9	0.81	7	14	0.99	5	9	
	Makobe	0.88	3	5	0.94	5	10	0.97	8	18	0.92	5	10	
	Ruti	NA	1	2	0.81	8	17	0.86	6	11	NA	1	2	
	All islands	0.91	13	25	0.86	18	38	0.93	26	52	0.82	18	34	
anal fin spot	Kissenda	NA	1	4	NA	1	1	-0.10	5	10	0.64	7	14	
	Python	0.94	8	14	0.41	4	8	0.90	7	12	0.36	5	10	
	Makobe	0.89	10	19	0.74	5	14	0.93	9	19	0.85	5	10	
	Ruti	0.88	11	21	0.95	8	16	0.75	6	14	NA	1	2	
	All islands	0.89	30	58	0.78	18	39	0.79	27	55	0.82	18	36	
flank	Kissenda	NA	1	2	NA	1	1	0.88	5	11	0.49	7	14	
	Python	0.96	9	19	0.37	5	19	0.93	7	16	0.99	5	10	
	Makobe	0.82	10	20	0.64	5	12	0.93	9	21	0.59	5	12	
	Ruti	0.92	11	21	0.77	8	16	0.79	6	16	NA	1	2	
	All islands	0.92	31	62	0.71	19	48	0.92	27	64	0.73	18	38	

Supplementary Table S3: Repeatability for reflectance data: λP_{50}

		light source: lamp						light source: sun					
		P. nyererei			*P. pundamilia*			*P. nyererei*			*P. pundamilia*		
		r	N fish	N spectra	r	N fish	N spectra	r	N fish	N spectra	r	N fish	N spectra
dorsum	Kissenda	NA	1	2	NA	1	2	0.99	5	10	0.76	7	15
	Python	0.95	9	21	0.92	5	13	0.91	7	13	0.18	5	10
	Makobe	0.39	9	22	0.73	5	10	0.91	9	20	0.93	4	8
	Ruti	0.98	11	24	0.90	8	16	0.74	6	15	NA	1	2
	All islands	0.90	30	69	0.85	19	41	0.94	27	58	0.75	17	35
dorsal fin	Kissenda	NA	1	2	NA	1	2	0.73	5	9	0.43	7	14
	Python	0.87	9	20	0.78	4	12	0.72	7	13	0.61	5	10
	Makobe	0.94	9	18	0.89	5	12	0.85	7	15	0.86	5	9
	Ruti	0.92	11	22	0.99	8	16	0.93	6	14	NA	1	2
	All islands	0.97	30	62	0.91	18	42	0.93	25	51	0.71	18	35
dorsal fin lappets	Kissenda	NA	1	2	NA	1	2	0.51	5	9	0.55	7	13
	Python	0.85	8	16	0.85	4	9	0.81	7	14	0.98	5	9
	Makobe	0.93	3	5	0.51	5	10	0.96	8	18	0.92	5	10
	Ruti	NA	1	2	0.84	8	17	0.80	6	11	NA	1	2
	All islands	0.87	13	25	0.76	18	38	0.82	26	52	0.83	18	34
anal fin spot	Kissenda	NA	1	4	NA	1	1	-0.18	5	10	0.82	7	14
	Python	0.99	8	14	-0.60	4	8	0.91	7	12	0.72	5	10
	Makobe	0.93	10	19	0.75	5	14	0.92	9	19	0.91	5	10
	Ruti	0.79	11	21	0.97	8	16	0.83	6	14	NA	1	2
	All islands	0.93	30	58	0.83	18	39	0.83	27	55	0.90	18	36
flank	Kissenda	NA	1	2	NA	1	1	0.88	5	11	0.65	7	14
	Python	0.98	9	19	0.60	5	19	0.93	7	16	0.98	5	10
	Makobe	0.82	10	20	0.75	5	12	0.89	9	21	-0.54	5	12
	Ruti	0.93	11	21	0.91	8	16	0.83	6	16	NA	1	2
	All islands	0.96	31	62	0.85	19	48	0.93	27	64	0.45	18	38

Supplementary Table S4: Spearman correlations between body areas for chroma														
		light source: lamp						light source: sun						
		P. nyererei			*P. pundamilia*			*P. nyererei*			*P. pundamilia*			
		rho	p	N	rho	p	N	rho	p	N	rho	p	N	
dorsal fin lappets-anal fin spot	Kissenda	NA	NA	1	NA	NA	1	-0.10	0.95	5	0.64	0.14	7	
	Python	0.50	0.22	8	0.50	1.00	3	-0.07	0.91	7	0.50	0.45	5	
	Makobe	0.50	1.00	3	-0.14	0.80	6	-0.05	0.93	8	0.70	0.23	5	
	Ruti	NA	NA	1	0.12	0.79	8	0.54	0.30	6	NA	NA	1	
dorsal fin lappets-flank	Kissenda	NA	NA	1	NA	NA	1	-0.40	0.52	5	-0.68	0.11	7	
	Python	-0.29	0.50	8	-0.40	0.75	4	-0.32	0.50	7	-0.30	0.68	5	
	Makobe	0.50	1.00	3	-0.09	0.92	6	-0.26	0.54	8	0.50	0.45	5	
	Ruti	NA	NA	1	-0.17	0.70	8	-0.09	0.92	6	NA	NA	1	
dorsal fin-dorsal fin lappets	Kissenda	NA	NA	1	NA	NA	1	-0.90	0.08	5	-0.32	0.50	7	
	Python	0.33	0.43	8	-0.80	0.33	4	0.07	0.91	7	0.30	0.68	5	
	Makobe	-1.00	1.00	2	-0.26	0.66	6	-0.32	0.50	7	0.20	0.78	5	
	Ruti	NA	NA	1	0.33	0.43	8	0.66	0.18	6	NA	NA	1	
dorsal fin-dorsum	Kissenda	NA	NA	1	NA	NA	1	0.80	0.13	5	0.00	1.00	7	
	Python	0.20	0.61	9	-0.20	0.92	4	0.07	0.91	7	0.60	0.35	5	
	Makobe	0.52	0.20	8	0.60	0.24	6	-0.25	0.59	7	0.40	0.75	4	
	Ruti	0.72	**0.04**	9	0.45	0.27	8	0.26	0.66	6	NA	NA	1	
dorsal fin-anal fin spot	Kissenda	NA	NA	1	NA	NA	1	0.00	1.00	5	-0.25	0.59	7	
	Python	0.36	0.39	8	-1.00	0.33	3	0.75	0.07	7	0.90	0.08	5	
	Makobe	-0.65	0.07	9	-0.20	0.71	6	-0.68	0.11	7	-0.30	0.68	5	
	Ruti	0.36	0.27	11	-0.40	0.33	8	0.71	0.14	6	NA	NA	1	
dorsal fin-flank	Kissenda	NA	NA	1	NA	NA	1	0.30	0.68	5	0.32	0.50	7	
	Python	-0.07	0.88	9	0.80	0.33	4	-0.14	0.78	7	0.50	0.45	5	
	Makobe	0.22	0.58	9	0.83	0.06	6	0.04	0.96	7	0.90	0.08	5	
	Ruti	0.67	**0.03**	11	0.40	0.33	8	-0.09	0.92	6	NA	NA	1	
dorsum-dorsal fin lappets	Kissenda	NA	NA	1	NA	NA	1	-0.50	0.45	5	-0.61	0.17	7	
	Python	0.14	0.75	8	0.40	0.75	4	-0.61	0.17	7	-0.10	0.95	5	
	Makobe	1.00	1.00	2	-0.43	0.42	6	0.19	0.66	8	-0.40	0.75	4	
	Ruti	NA	NA	1	0.19	0.66	8	0.26	0.66	6	NA	NA	1	
dorsum-anal fin spot	Kissenda	NA	NA	1	NA	NA	1	-0.30	0.68	5	-0.61	0.17	7	
	Python	-0.50	0.22	8	-0.80	0.33	4	-0.07	0.91	7	0.70	0.23	5	
	Makobe	-0.82	**0.01**	9	0.43	0.42	6	-0.35	0.36	9	-0.40	0.75	4	
	Ruti	0.33	0.39	9	-0.40	0.33	8	-0.09	0.92	6	NA	NA	1	
dorsum-flank	Kissenda	NA	NA	1	NA	NA	1	0.30	0.68	5	0.50	0.27	7	
	Python	0.20	0.61	9	0.50	0.45	5	0.43	0.35	7	0.90	0.08	5	
	Makobe	0.07	0.88	9	0.20	0.71	6	0.17	0.68	9	-0.20	0.92	4	
	Ruti	0.27	0.49	9	0.00	1.00	8	0.77	0.10	6	NA	NA	1	
anal fin spot-flank	Kissenda	NA	NA	1	NA	NA	1	0.20	0.78	5	-0.57	0.20	7	
	Python	-0.26	0.54	8	-1.00	0.08	4	0.14	0.78	7	0.60	0.35	5	
	Makobe	0.07	0.86	10	-0.66	0.18	6	-0.45	0.23	9	-0.10	0.95	5	
	Ruti	0.54	0.09	11	-0.86	**0.01**	8	0.03	1.00	6	NA	NA	1	

Supplementary Table S5: Spearman correlations between body areas for λP_{50}													
		light source: lamp						light source: sun					
		P. nyererei			*P. pundamilia*			*P. nyererei*			*P. pundamilia*		
		rho	p	N	rho	p	N	rho	p	N	rho	p	N
dorsal fin lappets-anal fin spot	Kissenda	NA	NA	1	NA	NA	1	-0.10	0.95	5	0.63	0.13	7
	Python	0.36	0.39	8	0.50	1.00	3	-0.07	0.91	7	0.50	0.45	5
	Makobe	0.87	0.33	3	0.03	1.00	6	-0.06	0.89	8	0.70	0.23	5
	Ruti	NA	NA	1	-0.02	0.95	8	0.54	0.30	6	NA	NA	1
dorsal fin lappets-flank	Kissenda	NA	NA	1	NA	NA	1	-0.20	0.78	5	-0.04	0.96	7
	Python	0.00	1.00	8	0.40	0.75	4	-0.32	0.50	7	0.30	0.68	5
	Makobe	0.87	0.33	3	-0.37	0.50	6	-0.24	0.57	8	-0.41	0.49	5
	Ruti	NA	NA	1	-0.61	0.11	8	-0.09	0.92	6	NA	NA	1
dorsal fin-dorsal fin edge	Kissenda	NA	NA	1	NA	NA	1	-0.70	0.23	5	0.29	0.56	7
	Python	-0.19	0.66	8	0.40	0.75	4	0.34	0.45	7	-0.30	0.68	5
	Makobe	-1.00	1.00	2	-0.12	0.83	6	-0.32	0.50	7	0.40	0.52	5
	Ruti	NA	NA	1	-0.30	0.47	8	0.66	0.18	6	NA	NA	1
dorsal fin-dorsum	Kissenda	NA	NA	1	NA	NA	1	0.90	0.08	5	0.89	0.01	7
	Python	0.57	0.12	9	-0.40	0.75	4	-0.27	0.56	7	0.90	0.08	5
	Makobe	0.48	0.24	8	0.67	0.15	6	-0.25	0.59	7	0.80	0.33	4
	Ruti	0.67	**0.05**	9	0.86	0.01	8	0.26	0.66	6	NA	NA	1
dorsal fin-anal fin spot	Kissenda	NA	NA	1	NA	NA	1	0.10	0.95	5	0.31	0.50	7
	Python	-0.55	0.17	8	0.50	1.00	3	0.49	0.27	7	-0.90	0.08	5
	Makobe	-0.58	0.11	9	0.52	0.29	6	-0.65	0.11	7	0.10	0.95	5
	Ruti	0.50	0.12	11	0.59	0.13	8	0.71	0.14	6	NA	NA	1
dorsal fin-flank	Kissenda	NA	NA	1	NA	NA	1	0.20	0.78	5	0.46	0.30	7
	Python	0.25	0.52	9	0.60	0.42	4	0.23	0.61	7	0.50	0.45	5
	Makobe	-0.08	0.85	9	0.90	0.01	6	0.05	0.91	7	0.41	0.49	5
	Ruti	0.21	**0.54**	11	0.60	0.13	8	-0.09	0.92	6	NA	NA	1
dorsum-dorsal fin lappets	Kissenda	NA	NA	1	NA	NA	1	-0.50	0.45	5	0.04	0.96	7
	Python	-0.38	0.36	8	-0.40	0.75	4	-0.61	0.17	7	0.10	0.95	5
	Makobe	NA	NA	2	-0.09	0.92	6	0.24	0.57	8	0.20	0.92	4
	Ruti	NA	NA	1	-0.19	0.65	8	0.26	0.66	6	NA	NA	1
dorsum-anal fin spot	Kissenda	NA	NA	1	NA	NA	1	-0.30	0.68	5	0.41	0.36	7
	Python	-0.36	0.39	8	0.40	0.75	4	-0.07	0.91	7	-0.70	0.23	5
	Makobe	-0.92	**0.00**	9	-0.20	0.71	6	-0.44	0.24	9	0.20	0.92	4
	Ruti	0.54	0.13	9	0.54	0.17	8	-0.09	0.92	6	NA	NA	1
dorsum-flank	Kissenda	NA	NA	1	NA	NA	1	-0.10	0.95	5	0.71	0.09	7
	Python	0.67	0.06	9	0.36	0.55	5	0.43	0.35	7	0.60	0.35	5
	Makobe	0.11	0.78	9	0.83	0.06	6	-0.03	0.93	9	0.80	0.33	4
	Ruti	0.10	0.80	9	0.42	0.30	8	0.77	0.10	6	NA	NA	1
anal fin spot-flank	Kissenda	NA	NA	1	NA	NA	1	0.50	0.45	5	0.45	0.31	7
	Python	0.12	0.79	8	0.95	0.05	4	-0.21	0.66	7	-0.60	0.35	5
	Makobe	0.41	0.24	10	0.20	0.71	6	-0.36	0.34	9	-0.67	0.22	5
	Ruti	0.38	0.25	11	0.12	**0.78**	8	0.03	1.00	6	NA	NA	1

Supplementary Table S6: descriptive statistics for the effect of using different light sources

colour metric	species	body area	light source<br>lamp (mean ± se, N)	 sunlight (mean ± se, N)
Brightness	*P. nyererei*	dorsum	646.08 ± 67.85 (28)	2897.22 ± 402.58 (27)
		dorsal fin	1982.62 ± 157.26 (32)	6265.16 ± 759.82 (25)
		dorsal fin lappets	1141.35 ± 163.4 (13)	5770.37 ± 920.09 (26)
		anal fin spot	2401.1 ± 159.56 (30)	5963.13 ± 810.37 (27)
		flank	1934.79 ± 150.32 (31)	5903.28 ± 685.93 (27)
	P. pundamilia	dorsum	642.07 ± 156.46 (20)	2253.07 ± 359.62 (17)
		dorsal fin	1611.3 ± 142.9 (19)	4402.05 ± 734.25 (18)
		dorsal fin lappets	767.62 ± 141.32 (19)	4282.96 ± 933.16 (18)
		anal fin spot	1498.26 ± 134.5 (19)	6187.43 ± 824.95 (18)
		flank	1796.6 ± 222.97 (20)	5082.14 ± 563.47 (18)
Chroma	*P. nyererei*	dorsum	0.3 ± 0.03 (28)	0.28 ± 0.03 (27)
		dorsal fin	0.4 ± 0.04 (32)	0.24 ± 0.04 (25)
		dorsal fin lappets	0.42 ± 0.04 (13)	0.27 ± 0.03 (26)
		anal fin spot	0.33 ± 0.02 (30)	0.15 ± 0.02 (27)
		flank	0.24 ± 0.02 (31)	0.19 ± 0.02 (27)
	P. pundamilia	dorsum	0.11 ± 0.02 (20)	0.09 ± 0.01 (17)
		dorsal fin	0.17 ± 0.02 (19)	0.1 ± 0.02 (18)
		dorsal fin lappets	0.38 ± 0.04 (19)	0.2 ± 0.02 (18)
		anal fin spot	0.39 ± 0.02 (19)	0.2 ± 0.02 (18)
		flank	0.17 ± 0.02 (20)	0.07 ± 0.01 (18)
λP_{50}	*P. nyererei*	dorsum	588.29 ± 8.26 (28)	603.37 ± 5.15 (27)
		dorsal fin	596.06 ± 9.36 (32)	591.04 ± 6.14 (25)
		dorsal fin lappets	617.62 ± 4.6 (13)	599.65 ± 4.36 (26)
		anal fin spot	605.17 ± 2.93 (30)	577.59 ± 3.35 (27)
		flank	572.19 ± 3.83 (31)	578.52 ± 3.73 (27)
	P. pundamilia	dorsum	536.95 ± 5.03 (20)	553.94 ± 4.51 (17)
		dorsal fin	527.37 ± 4.24 (19)	535.72 ± 4.69 (18)
		dorsal fin lappets	613.74 ± 7.15 (19)	589.5 ± 5.07 (18)
		anal fin spot	615.32 ± 2.59 (19)	585.83 ± 4.33 (18)
		flank	531.5 ± 5.23 (20)	544.72 ± 3.93 (18)
